# Ion removal effect by soil remediation and desalination

**DOI:** 10.1371/journal.pone.0344492

**Published:** 2026-04-27

**Authors:** Chendi Shi, Haozhe Wang, Yuxuan Li, Mingxiang Xu, Xilian Luo

**Affiliations:** 1 China Shaanxi Well-Facilitated Farmland Construction Group Co., Ltd., Yangling, China; 2 Shaanxi Laboratory for Arid Agriculture, Northwest A&F University, Yangling, China; 3 School of Human Settlements and Civil Engineering, Xi’an Jiaotong University, Xi’an, China; 4 College of Soil and Water Conservation Science and Engineering (Institute of Soil and Water Conservation), Northwest A&F University, Yangling, China; Indian Institute of Technology Roorkee, INDIA

## Abstract

Electrokinetic remediation of saline soils currently faces two primary constraints: high energy consumption and considerable variability in the removal efficiency of salts and base ions under differing treatment conditions. These limitations have hindered the widespread practical adoption of this technology.This study explores the Ion removal effect by soil remediation and desalination. Through laboratory simulations, the base ion removal effects and energy consumption under various electric field conditions were investigated. The results show that electrokinetic remediation at 2.0 V/cm can effectively remove base ions from the soil, with notable removal efficiencies of Na^+^ and Cl^-^. It was also found that the electric field strength significantly affects both the remediation effect and energy consumption. An excessively high electric field strength may lead to a sharp decrease in soil moisture content, which in turn affects the remediation efficiency and increases energy consumption. Electrokinetic remediation has considerable potential for improving saline soils. By optimizing the electric field conditions and controlling pH levels, remediation can be enhanced while energy consumption is reduced. However, in practical applications, factors such as soil type and pollution degree must be considered, and further in-depth research is needed to refine and optimize remediation technology.

## 1. Introduction

**Fig 1:**
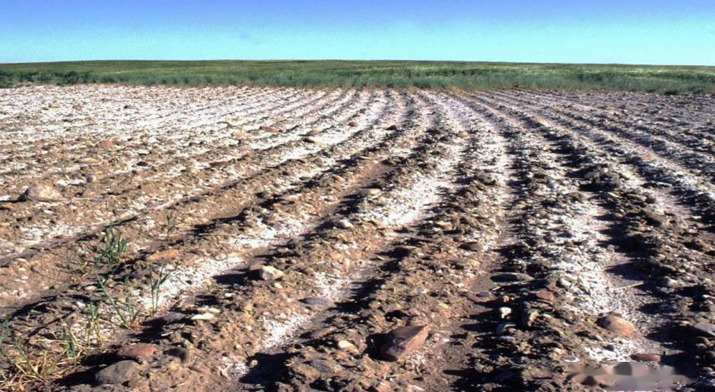
Photograph of soil salinization.

Soil salinization is the process in which soluble salt ions continuously accumulate at the soil surface (see Fig 1), changing the physical and chemical properties of soil leads to adverse changes in its basic characteristics, resulting in a decline in soil quality [1]. Currently, soil salinization is increasing globally. According to data from the Food and Agriculture Organization (FAO), approximately 83.3 million hectares of soil worldwide are under threat of salinization, with the highest concentrations observed in Eurasia, Africa, and the western regions of the Americas [2]. The total area of saline soils in China is approximately 36.9 million hectares, representing 5.01% of the nation’s arable land. These soils are predominantly distributed across the northeastern, northern, and northwestern regions, as well as the middle and lower reaches of the Yangtze River and coastal zones [3]. Saline soils are detrimental to plant growth, as they hinder the absorption of water and essential nutrients, potentially leading to plant death [4]. A high salt content negatively impacts soil structure, causing soil compaction, disruption of soil aggregates, and a reduction in soil aeration and water retention capacity [5]. Additionally, salts in saline soils can leach into groundwater or be carried by runoff into water bodies, resulting in water pollution and degrading water quality [6].

The amelioration and management of saline soils encompass a range of approaches, including physical, chemical, and biological methods [7,8]. Saline soil management is based on changing the upper and lower boundary conditions of the soil and adjusting the relevant soil‒water–air–biological parameters to regulate the movement and accumulation of soil salts [9]. For example, through water management, salts in the plow layer can be leached to form a “desalinated fertile layer” or localized “water and fertilizer retention layer” [10]. Water management can also involve reducing surface evaporation and crop transpiration to inhibit surface salt accumulation, creating loose impermeable layers to impede upward salt movement and thereby reducing soil permeability in certain layers, increasing soil drainage to accelerate salt leaching and improve overall soil permeability, or directing salt accumulation away from the root zone to avoid salinity [11].

As an innovative soil restoration method, electrokinetic remediation is based on the application of an electric field to induce the directional migration of salts, heavy metals, and other substances within the soil, thereby achieving soil remediation. The underlying principle is based on the migration behavior of ions in response to an electric field [12]. By controlling parameters such as voltage and current density, the direction and rate of ion migration can be effectively regulated. In recent years, extensive research has been conducted both domestically and internationally on the electrokinetic remediation of soil heavy metal ions and organic pollutants [12].

Common electrode materials include stainless steel and titanium alloys, among others. Wen et al. [13] used stainless steel and graphite as electrodes in electrokinetic remediation experiments and revealed that compared with graphite electrodes, stainless steel electrodes do not cause significant fluctuations in the remediation current, nor do they have a notable effect on the remediation effectiveness of heavy metals. However, during electrokinetic remediation, some background salts in the soil can precipitate, forming CaCO_3_ and Ca(OH)_2_ crystals near the cathode. This phenomenon leads to a decrease in the conductivity of stainless steel electrodes [14]. Suzuki et al. [15] investigated the efficiency of electrokinetic remediation for lead removal using graphite, Pt/Ti, and IrO_2_/Ti as anodes. The removal efficiency decreased in the following order: Pt/Ti, IrO_2_/Ti, and graphite. Méndez et al. [16] found that the passivation of electrode materials such as stainless steel and titanium leads to a decrease in surface activity and an increase in resistance. In contrast, carbon electrodes possess excellent chemical stability, electrical conductivity, and cost-effectiveness, as well as strong adsorption capacity for organic pollutants, making them highly promising for application in electrokinetic remediation of soils, especially suitable for treating complex mixtures of contaminants. Velizarova et al. [17] concluded that the migration rates of ions such as copper and arsenic do not exhibit a linear correlation with the applied current intensity.

In the process of electrokinetic remediation, soil pH directly influences the chemical form, solubility, and mobility of contaminants. Villen-Guzman et al. [18] reported that the use of sodium ethylenediaminetetraacetate (NaEDTA) and citric acid (CA) to control the pH of the working solution is beneficial for increasing soil remediation efficiency. Li et al. [19] used citric acid as the cathodic electrolyte and employed an anode approximation approach to remediate heavy metal-contaminated soil. This method changed the binding forms of ions with the soil, thereby enhancing the ion removal efficiency. Vizcaino et al. [20] explored the effects of electrokinetic remediation and found that adding NaOH solution near the anode could enhance the removal efficiency of contaminants by controlling the local pH. Ryu et al. [21] investigated the use of acidic and alkaline solutions for soil pretreatment and electrolyte adjustment. The study revealed that nitric acid enhanced the removal rates of Cu and Pb from soil, while the use of sodium hydroxide to adjust the anolyte improved the removal efficiency of As. Cang et al. [22] utilized a buffer solution to control the pH of the anolyte, raising the soil pH to 12, which significantly improved the removal rates of Cr(III) and Cr(VI). Zhou et al. [23] found that controlling soil acidity with lactic acid and citric acid, along with the addition of ethylenediaminetetraacetate acid(EDTA) for complexation, effectively reduced chromium levels in yellow‒brown soil. Qian et al. [24] used an appropriate amount of citric acid as a washing solution in the remediation of Cu-contaminated soil, and the results demonstrated that under suitable conditions, the removal rate of Cu could reach 89.9%. Peng and Tian [25] found that soil treated with citric acid achieved high removal efficiencies for chromium and zinc, while the improvement in the removal efficiency of copper and lead was relatively low.

During electrokinetic remediation, hydrolysis reactions occurring near the electrodes generate H⁺ and OH⁻ ions, which can directly influence remediation efficiency. Darmawan and Wada [26] employed cation exchange resins between the soil chamber and the cathode to prevent excessive OH⁻ from entering the soil and to capture positively charged heavy metal ions that had migrated. Li et al. [27] designed a salt solution zone between the soil chamber and the cathode electrode plate with incorporated cation exchange membranes. This setup allowed heavy metals to migrate out of the soil and accumulate in the solution rather than precipitating back into the soil. The copper (Cu) removal rate reached 90%. Song et al. [28] integrated dual cation exchange membranes and a recirculation method to enhance the effectiveness of EDTA in electrodynamic soil remediation. Ma et al. [29] incorporated cation-selective membranes and anion-selective membranes into a remediation system, and the experimental results demonstrated a significant improvement in remediation efficiency.

The cost of electrokinetic remediation is one of the primary factors limiting its widespread application. Energy consumption in electrokinetic remediation can be reduced by using renewable energy sources such as solar power to replace traditional electricity supplies, and by applying pulsed direct current (PDC) or intermittent power to mitigate thermal effects.

Alshawabkeh et al. [30] noted that energy consumption accounts for 16.1% of the total cost of electrokinetic remediation. Jeon et al. [31] demonstrated that high ionic strength in soil leads to increased soil conductivity, thereby resulting in increased energy consumption. Yuan et al. [32] conducted research on solar energy as an alternative to electricity for electroremediation. Hassan et al. [33] and Jeon et al. [31] also utilized solar energy for electroremediation and achieved satisfactory results. Furthermore, Yuan et al. [34] employed a galvanic cell as a power source, and realized successful electroremediation of copper-contaminated soil. In electroremediation, stabilized voltage or current supply methods are commonly used. Studies have shown that the voltage gradient has a significant effect on electroosmosis, and the soil chemical changes induced by different voltage gradients also notably influence electroosmotic flow [35]. The use of a pulsed power supply in electroremediation can reduce the adverse effects caused by polarization. Kim et al. [36] discovered that pulsed voltage electroremediation cannot only reduce energy consumption but also regulate soil pH and soil conductivity while effectively preventing electrode corrosion. Jo et al. [37] applied pulsed voltage electroremediation to remove sulfate, nitrate, potassium, sodium, and other salt ions from soil and achieved effective results. Lee et al. [38] conducted field experiments using pulsed voltage electroremediation, and the results demonstrated that pulsed voltage electroremediation treatment of outdoor saline‒alkali soils was significantly effective.

Although electrokinetic remediation has been widely applied in the treatment of heavy metal- and organic-contaminated soils, its application to saline soils remains relatively underexplored. Most existing studies focus on heavy metal migration and pH adjustment, while only a limited number of works have addressed the behavior and removal efficiency of base ions such as Na^+^, Cl^-^, and SO_4_^2-^ in artificially or naturally saline soils. Moreover, studies systematically investigating the impact of different electric field strengths on ion removal and energy consumption in saline soil remediation are particularly scarce. This gap highlights the need for targeted research to optimize EKR parameters for saline soil applications. Further attention is needed regarding the following issues:

(1) This study fills the gap in the study of the removal rate and energy consumption of basic ions (Na ⁺ , Cl⁻ and SO₄²⁻) with time under different electric field intensities in saline-alkali soil.
(2) The effects of electric fields on the removal efficiency and energy consumption of base ions in saline soils under different remediation conditions.
(3) The optimal electric field conditions and remediation duration for electrokinetic remediation of saline soils under different treatment conditions.

This study conducted electrokinetic remediation of saline soils under various electric field conditions and dynamically monitored the removal of base ions with the aim of improving the efficiency of electrokinetic remediation, reducing remediation costs, and minimizing secondary pollution. The main contribution of this research are as follows:

(1) The study revealed the effects of different electric field conditions on the removal efficiency of base ions and energy consumption, and identified the optimal remediation conditions and duration.
(2) The feasibility of electrokinetic remediation technology for saline soil treatment was verified, providing technical support for its application in saline soil remediation.

## 2. Materials and methods

### 2.1 Experimental principle

**Fig 2:**
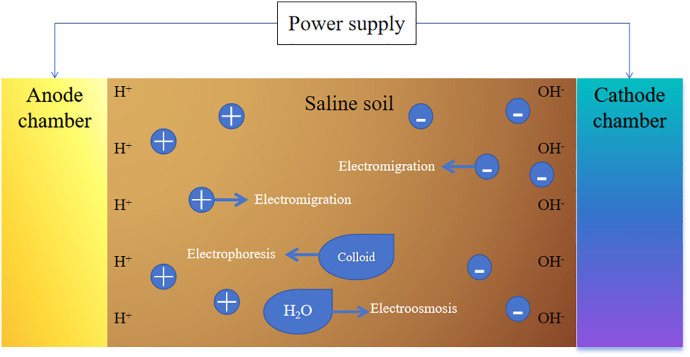
Schematic diagram of the principle of electrokinetic remediation of contaminated soil.


Tombuloglu et al. [39] conducted extensive research on the principles and influencing factors of electrokinetic remediation. As shown in Fig 2, The primary mechanism involves the application of an external power source to provide an electric potential, which facilitates electromigration, electroosmosis, and electrophoresis, thereby influencing ion migration. In electrokinetic remediation, pollutant migration is affected by multiple electrokinetic effects and is also closely related to factors such as soil conductivity [40]. In most cases, electromigration is the primary ion transport mechanism. During electrokinetic remediation, the hydrolysis reactions at the electrodes play a crucial role in ion migration.

$$\text{2}H_{\text{2}}O\to \text{4}{\text{e}}^{-}+O_{\text{2}}+\text{4}H^{+}\;(A\text{node})$$ (1)

$$\text{4}H_{\text{2}}O+\text{4}{\text{e}}^{-}\to {\text{2}H}_{\text{2}}+\text{4}{OH}^{-}\;(C\text{athode})$$ (2)

Electromigration refers to the directional movement of ions or ion complexes in soil or sediments under the influence of electrokinetic forces. When a large number of salt ions are present in the soil pore water, significant migration occurs. The rate of electromigration is primarily influenced by factors such as ion concentration, temperature, voltage intensity, ion charge, ionic concentration, conductivity, temperature, and pH. Electroosmosis refers to the movement of soil pore water relative to the soil under electrokinetic forces [41]. In contrast, electrophoresis, in the context of electrokinetic remediation, refers to the directional migration of charged colloidal particles in an electric field. Colloidal particles generally include fine soil particles, microorganisms, humus, and other substances. Compared with electromigration and electroosmosis, electrophoresis plays a relatively minor role in electrokinetic remediation. The salts in saline soils essentially exist in the form of dissolved ions, making them typical targets for electromigration. Electromigration directly drives these ions to migrate toward the corresponding electrodes, and is therefore the primary driving mechanism for salt removal. Although electroosmosis contributes to the transport of pore water and some neutral molecules, it plays a more auxiliary role in the migration and removal of salt-base ions. This is especially true in low-permeability soils, where the electroosmotic flow rate is limited and its contribution is relatively minor. Electrophoresis only affects colloidal particles or macromolecules such as humus and microorganisms, and does not directly influence the migration of dissolved ions; thus, its role in salt remediation is negligible [42].

### 2.2 Experimental system

**Fig 3:**
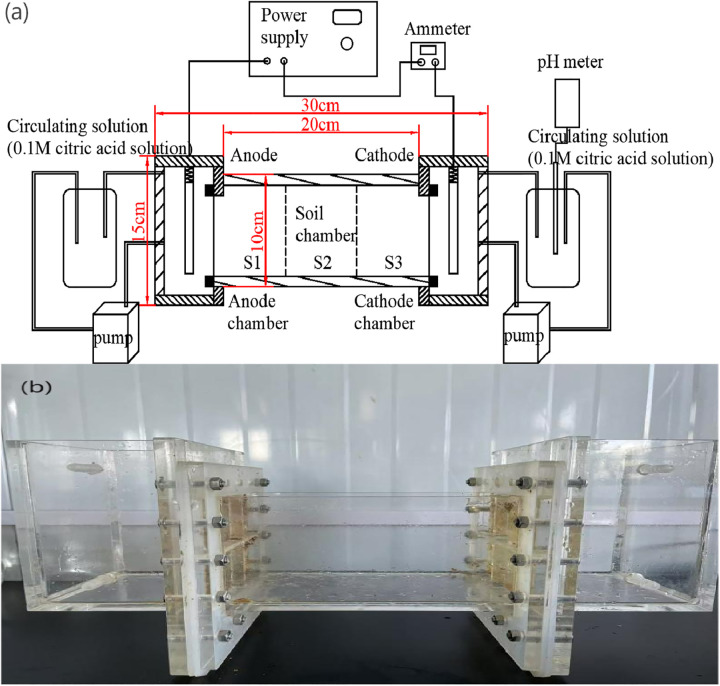
Schematic diagram (a) and photograph of the electrokinetic soil remediation experimental device (b).

The design of the experimental apparatus is shown in Fig 3(a). After the three replicate soil samples treated for salinization were mixed, the mixture was placed into the experimental electrokinetic remediation device (Fig 3(b)), with a soil mass of approximately 1.8 kg. During soil loading, 45.7 g of distilled water was added for every 0.3 kg of soil using a sprayer, maintaining a soil moisture content of 20%. The soil samples were left to stand for 24 h to allow uniform distribution of moisture within the soil. Based on the particle size analysis, the soil consisted of 24.20% sand, 71.20% silt, and 4.60% clay, classifying it as a silt loam according to the USDA soil texture triangle.

The experimental device is shown in Fig 3(a). It consisted of a self-designed organic glass working chamber (30 cm × 15 cm × 15 cm), which included three parts: a central soil chamber, anode and cathode electrode chambers at both ends, and a circulating electrolyte chamber. A DC adjustable voltage source with a range of 0 V/cm – 3.0 V/cm was used. The anode electrode was made of a 2 mm thick titanium plate, and the cathode electrode was made of a 2 mm thick stainless steel plate. The electrode dimensions were consistent with the cross-sectional area of the soil chamber, which measured 20 cm × 15 cm × 15 cm. The electrodes were placed 20 cm apart and fixed in the electrode chambers. The anode chamber (1000 mL) and the cathode chamber (1000 mL) were connected at both ends of the soil chamber (20 cm × 10 cm × 10 cm), with the electrode distance adjusted based on the electric field strength. The pH of the circulating solution was measured using a pH meter. A peristaltic pump was used to connect the electrode chambers and the circulating electrolyte chamber. The electrolyte solution used was 0.1 M citric acid solution. The electric field strengths were set at 1.0 V/cm, 2.0 V/cm, and 3.0 V/cm, with the environmental temperature maintained at 20°C ± 5°C.

### 2.3 Soil samples

The soil used in this experiment was collected from farmland in a village in Yangling District, Xianyang City, Shaanxi Province. The soil type is brown soil, with a loam texture(USDA). As measured in the experiment, the contents of sodium ion (Na^+^), chloride ion (Cl^-^), and sulfate ion (SO_4_^2-^) in the soil were 85 mg/kg, 133 mg/kg, and 140 mg/kg, respectively, and the soil pH was 8.62. After air-drying, grinding, and sieving, the soil samples were analyzed for Na^+^, Cl^-^, and SO_4_^2-^ contents using ion chromatography (IC). The soil pH was measured with a pH meter using a soil-to-distilled water mixture at a ratio of 1:2.5 (w/v). The sampling method involved removing surface debris, then using a spade to collect surface soil (0 cm – 20 cm depth) and placing it in clean plastic bags with appropriate labels. After collection, the soil was spread on a clean plastic sheet and left indoors to air dry naturally at a relative humidity of approximately 40%. After drying, plant fragments and large debris particles were removed, and the soil was crushed with a wooden rod and then ground with a ceramic mortar. The soil was sieved through 20- and 100-mesh screens and stored in sealed bags for further use.

The saline soil used in the experiment was prepared in the laboratory. The soil was subjected to salinization treatment, and after passing through a 100-mesh sieve, the soil samples were placed into open plastic boxes, with each box containing 20 kg of soil. The procedure was repeated three times. A graduated cylinder was used to add the prepared salt solution. The electric field repair conditions shown in Table 1. After mixing, the soil was air-dried, and the dried samples were passed through a 100-mesh sieve before use.

**Table 1:** Experimental treatments.

Experiment number	Remediation conditions	Distance	Total voltage
T1	1.0 V/cm	20 cm	20V
T2	2.0 V/cm	20 cm	40V
T3	3.0 V/cm	20 cm	60V

This study was conducted using severely salinized soil, as defined by the Chinese national standard for soil salinization (see Table 2). The mass fractions of Na^+^, Cl^-^, and SO_4_^2-^ in the saline soil were approximately 1500 mg/kg, 1000 mg/kg, and 4000 mg/kg, respectively, with a moisture content of 7%. The actual results after settling were used for sample preparation, and the ion concentrations were obtained from relevant studies.

**Table 2:** Standards for soil salinization in China.

Salinization standards	Total soluble salt content in soil (g/kg)
Slight salinization	1.0-3.0
Moderate salinization	3.0-5.0
Severe salinization	5.0-10.0
Saline soil	>10.0

A total of three experiments, denoted T1, T2, and T3, were conducted under different conditions. The voltage gradients were set to 1.0 V/cm, 2.0 V/cm, and 3.0 V/cm (see Table 1), corresponding to repair voltages of 20 V, 40 V, and 60 V, respectively. Each experiment included three sampling points, S1, S2, and S3, which were located at the midpoint of each third of the soil tank, with a distance of 6.7 cm between each sampling point.

### 2.4 Sample collection and analysis

During the electrokinetic remediation process, 10 g soil samples were collected every 12 h over a total period of 120 h, and the power consumption was recorded. After 120 h of electrokinetic treatment, the electrolyte solution was collected, and the pH of both the soil and electrolyte solution was measured using a pH meter.

The soil samples were placed in glass beakers, which were sealed with filter paper. The samples were then dried in an oven at 105°C until the soil weight no longer changes. After drying, the soil was ground and passed through 20-mesh and 100-mesh nylon sieves. The samples were stored in sealed plastic bags for further use. The concentrations of Na^+^, Cl^-^, and SO_4_^2-^ in the soil were measured via ion chromatography.

The formula for calculating the ion removal efficiency is as follows:


$$\omega_{\text{0}}=\frac{C_{\text{0}}-C_{\text{1}}}{C_{\text{0}}}\times \text{100}\%$$ (3)

where ω_0_ represents the ion removal rate, C₀ is the initial ion concentration in the soil (mg·kg^-1^), and C_1_ is the ion concentration remaining in the soil after electrokinetic remediation (mg·kg^-1^).

## 3. Results and analysis

### 3.1 Changes in soil and electrolyte pH

The initial pH of the soil was 8.62, and the initial pH of the electrolyte solution was 2.12. After 120 h of electrokinetic treatment, the pH values of the soil and electrolyte solution were measured. The changes in the pH values of the soil and electrolyte solution after electrokinetic remediation are shown in Table 3.

**Table 3:** Changes in soil and electrolyte pH.

Remediation conditions	Electrolyte pH at the anode	Electrolyte pH at the cathode	S1 pH	S2 pH	S3 pH
1.0 V/cm	2.05	10.87	4.92	6.45	10.25
2.0 V/cm	2.04	11.27	4.88	6.12	10.67
3.0 V/cm	2.07	11.03	5.31	6.67	9.81

**Fig 4:**
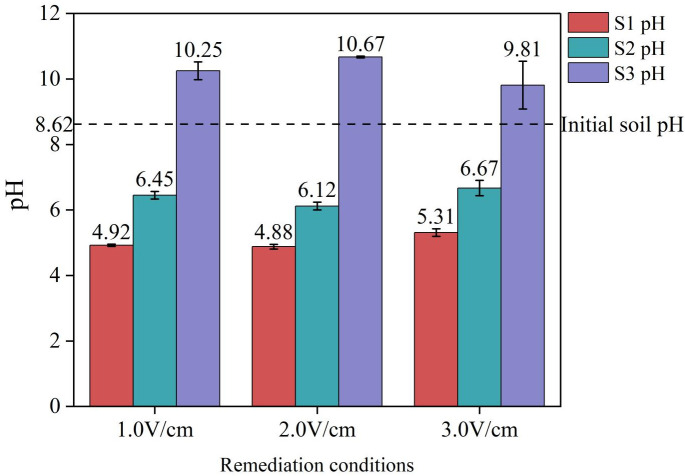
Changes in soil pH before and after treatment.

**Fig 5:**
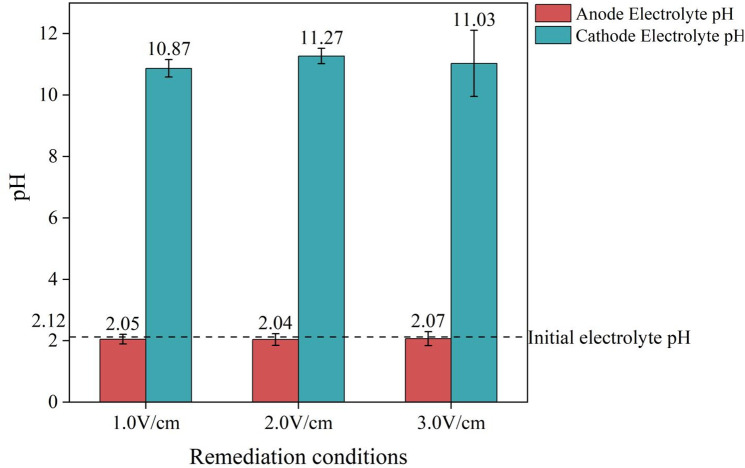
Changes in electrolyte pH before and after treatment.

As shown in Fig 4, the initial soil pH was 8.62. After electrokinetic remediation, the pH in region S1 decreased to 4.92, 4.88, and 5.31; in region S2, it decreased to 6.45, 6.12, and 6.67; while in region S3, the pH increased to 10.25, 10.67, and 9.81. According to Fig 5, the initial pH of the electrolyte was 2.12. After electrokinetic treatment, the pH of the anolyte decreased slightly to around 2.05, while the pH of the catholyte increased to approximately 11. Under the influence of electrolysis and an electric field, H^+^ ions are generated at the anode and migrate toward the cathode, leading to a gradual decrease in soil pH. OH^-^ ions are generated at the cathode and migrate toward the anode, causing the soil pH to gradually increase. In the three experimental groups, the pH of the soil in the S1 and S2 regions decreased, whereas the pH in the S3 region significantly increased. This is mainly due to the migration of H^+^ ions generated at the anode toward the cathode, and the migration of OH^-^ ions generated at the cathode toward the anode region. It can be concluded that acidic and alkaline migration zones exist in the soil. Research has shown that changes in pH affect the electrokinetic remediation effect and that controlling the pH is beneficial for the migration of ions in the soil [43].

### 3.2 Influence of different electric field conditions on ion removal efficiency

**Fig 6:**
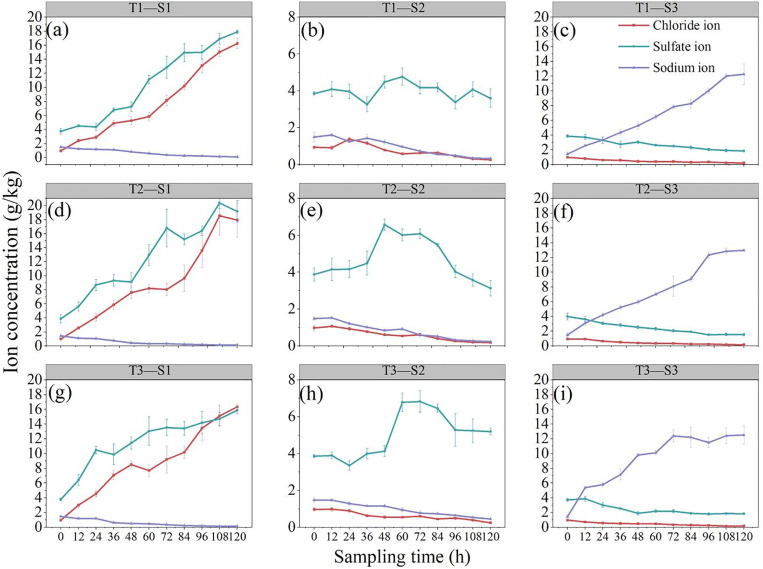
Changes over time in the concentrations of soil base ions at each sampling point under different conditions. Variation in base ion concentrations at different positions and treatments: **(a)** S1 in group T1; **(b)** S2 in group T1; **(c)** S3 in group T1; **(d)** S1 in group T2; **(e)** S2 in group T2; **(f)** S3 in group T2; **(g)** S1 in group T3; **(h)** S2 in group T3; **(i)** S3 in group T3.

Fig 6 shows that the first set of experimental salt ion concentrations were lower than the designed values. This occurred because during the moisture content control step, surface salt ions leached into the lower soil layer, resulting in an inflection point after the second or third sampling. During the experiment, there were cases of zero current under the conditions of 1.0 V/cm and 3.0 V/cm. This is because at 1.0 V/cm, the external electric field forces the “active” ions in the solution to migrate and act on the soil’s colloidal double layer system; these zero current events were also attributable to electrode polarization at both the anode and cathode. At 3.0 V/cm, the external electric field affects the soil’s colloidal charge balance system, leading to a sharp increase in soil resistance. At the same time, the adsorption energy between the colloidal core and ions is converted into thermal energy, causing the temperature of the soil system to rise sharply from 25 °C to 75 °C – 80 °C, further reducing the soil moisture content and thus decreasing the remediation effect.

The directional migration of ions in the soil under the action of an electric field is the main principle for saline soil improvement and electrokinetic remediation of contaminated soils. The migration and enrichment characteristics of ions are strongly related to their properties. After electrification, the Cl^-^ ions in all the experimental treatments were concentrated mainly near the anode, with a significant decrease in Cl^-^ content in the soil further from the anode. This is because Cl^-^ is not affected by soil base interactions and moves quickly under the influence of the electric field, resulting in a concentrated enrichment range, which facilitates removal. Near the anode, the Na^+^ content was low, while Na^+^ accumulated significantly near the cathode, indicating that at different voltages, Na^+^ migrates toward the cathode and concentrates there. There is a clear difference between the migration of anions and Na^+^ under an electric field. Anions migrate faster and concentrate in a smaller range near the anode, whereas Na^+^ migrates slower and has a slightly wider enrichment range. This is because Na^+^ has a positive charge and interacts with soil colloids, and under an electric field, its migration rate is significantly slower than that of anions.

A comparison of the changes in salt base ion concentrations at different sampling locations under the same electric field (Fig 6(a–c)) revealed that after electrification, the content of anions near the anode continuously increased, and the content of cations near the cathode also continuously increased. In treatment S2, the sulfate ion concentration increased slowly at first, then rapidly, with larger variations compared with those in the concentrations of other ions. This may be due to the slower overall migration rate of sulfate ions during the migration process; due to the migration of moisture, sulfate ions remain in the S2 region as they migrate from the cathode to the anode, resulting in a more dispersed enrichment range. This suggests that electrokinetic remediation has a lower removal effect on sulfate ions than other ions.

A comparison of the changes in ion concentrations under different treatment conditions at the same sampling location (Fig 6(a), 6(d) and 6(g)) revealed that at 2.0 V/cm, the remediation effect was more stable, with minor fluctuations and relatively high removal efficiency. Due to factors such as increased temperature and increased resistivity, the remediation effect under other conditions was unstable, and energy consumption tended to increase. After electrification, ions accumulated mainly near the electrodes. In the experiment, salt stains were observed in the soil near the electrodes, indicating that the rate of ion movement through the ion exchange membrane was slow and that some salt ions remained in the soil.

During the electrokinetic remediation of saline soils, the migration rate of base ions is a key parameter for evaluating the remediation effectiveness. This rate often exhibits nonlinear trends, accompanied by inflection points in the time dimension.

Based on the experimental data, it is evident that the concentration curves of the three ions at different soil profile positions display distinct phase-based variations. In particular, between 60 and 84 h of electrification, the migration rates generally transition from an ascending phase to a plateau or descending phase, indicating that the migration process has shifted from an active phase to a deceleration phase. The SO_4_^2-^ shows a distinct “mid-profile peak” characteristic in the S2 region, reaching a maximum between 60 and 72 h and slightly declining thereafter. This suggests that the ion initially accumulates in the mid-layer and subsequently migrates toward the anode to complete the migration process. In contrast, the Na^+^ concentration curve at the S3 position flattens between 72 and 96 h, indicating a declining migration rate over time.

**Fig 7:**
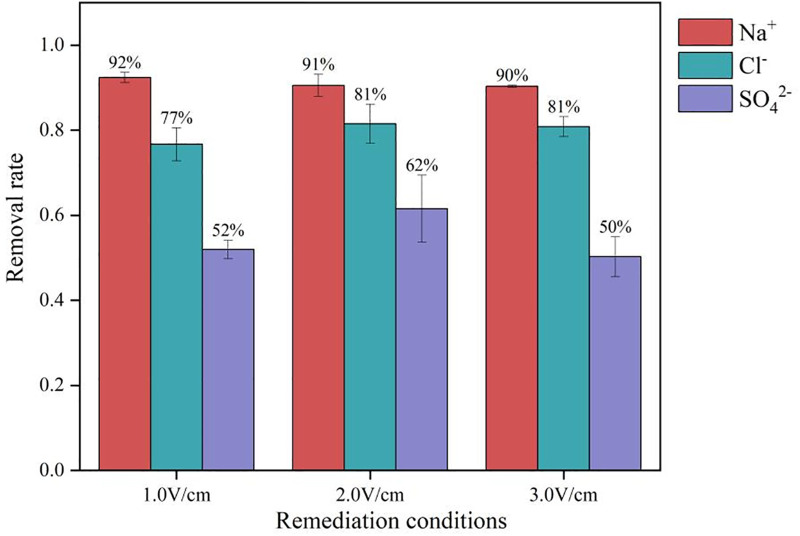
Ion removal efficiency between different treatments of soil remediation of saline soil.

**Fig 8:**
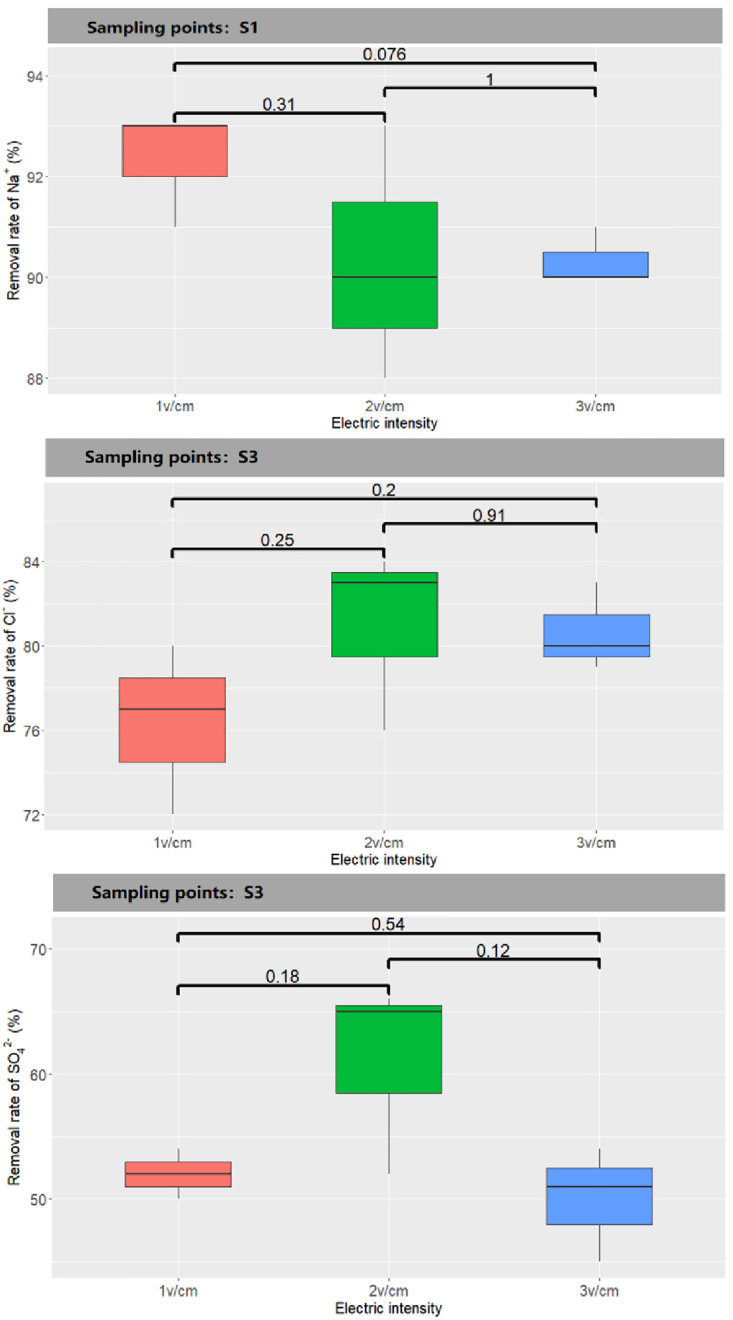
Significance test of ion removal efficiency between different treatment methods in soil remediation of saline soil.

The removal efficiency of salt base ions in the soil after electrokinetic treatment is shown in Fig 7. After electrokinetic remediation, the removal efficiency of Na^+^ was relatively high. The initial ω(Na^+^) of the soil was 1500 mg·kg^-1^. After remediation under the three electric fields, the remaining Na^+^ contents near S1 were 114.5, 134.4, and 142.4 mg·kg^-1^, with removal efficiencies of 92.41%, 90.56%, and 90.35%, respectively. Accumulation was observed near S2 and S3. Under the three treatments, the Cl^-^ contents near S3 were 228.5, 175.4, and 187.4 mg·kg^-1^, with removal efficiencies of 76.70%, 81.50%, and 80.86%, respectively. These values were slightly lower than the removal efficiency of Na^+^, but near S2, the Cl^-^ content was close to the content near S3, indicating that Cl^-^ migrates faster and that its enrichment range is smaller than that of Na^+^. This is because Na^+^ has a positive charge and adsorbs to soil colloids, so under an electric field, its migration rate is much slower than that of anions [44]. This finding indicates that electrokinetic remediation more easily removes anions than cations from the soil. The removal efficiency of sulfate ions was the lowest among the three ions. Under the three treatments, the removal efficiencies near S3 were 51.96%, 61.56%, and 50.28%.One-way analysis of t-tests was conducted to test the significance of differences among treatments. The analysis of the significance test (t-test) of the removal rates of different ions showed that there was no significant statistical difference between the removal rates of different ions. (Fig 8)

During the initial stage of soil remediation, many easily migratable anions and cations are present in the soil. At the start of soil remediation, the anode generates a large amount of H^+^ ions, which acidify the soil. Under acidic conditions, the binding force between metal ions or other charged ions on the surface of soil particles and the particles weakens, making the originally adsorbed ions more easily dissociate from the particle surfaces, resulting in a greater current in the soil. As electrokinetic remediation progresses, the current begins to decrease, which may be due to the migration of most of the charged particles in the soil toward areas near the electrodes [45].

### 3.3 Energy consumption of saline soil remediation under different electric field conditions

**Fig 9:**
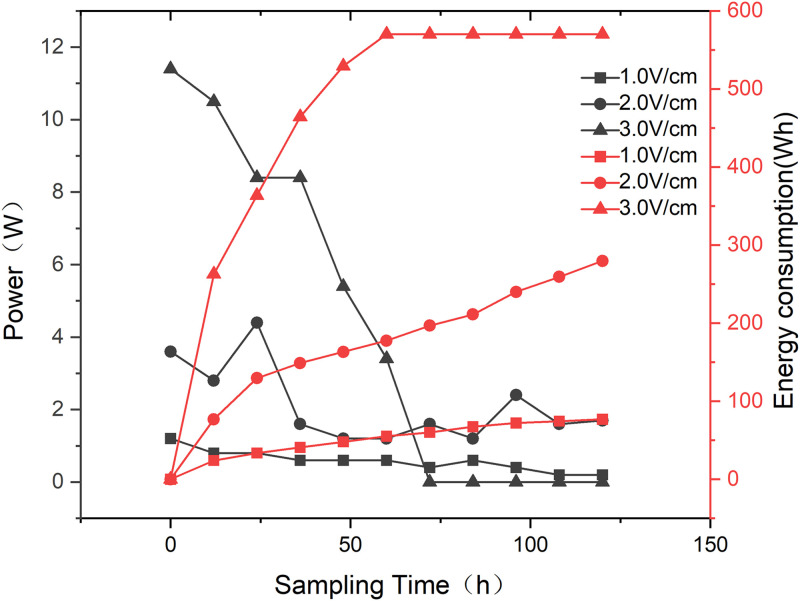
The variation of energy consumption with time and the total accumulated energy consumption during soil remediation.

**Fig 10:**
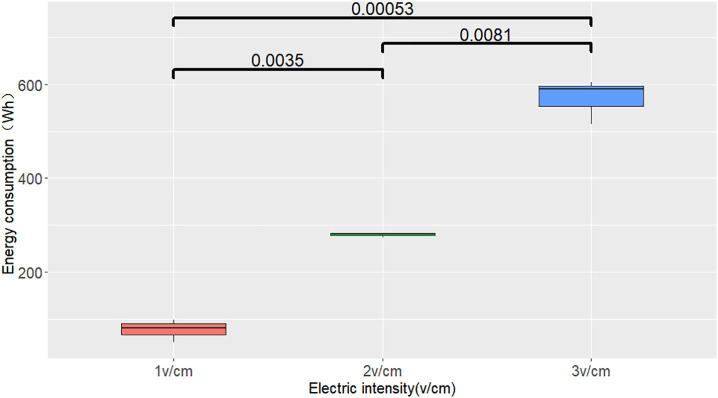
Significance test of total energy consumption and different treatment in soil temediation of saline soil.

Fig 9 presents the variation in power output during the soil remediation process, and depicts the corresponding changes in cumulative energy consumption. The energy consumption was the highest at 3.0 V/cm, but it reached zero after 72 h. This is likely because the high voltage causes water loss near the electrodes, leading to a rapid decrease in soil moisture. At 1.0 V/cm voltage conditions, energy consumption remained at its lowest level. The significance analysis of ion removal efficiency showed that compared with other treatments, the energy consumption of 1.0 V/cm treatment was lower and reached an extremely significant level (Fig 10). Energy consumption is based on the energy consumption of a certain volume of soil in the soil tank of the device

## 4. Discussion and conclusion

In constant-voltage electrokinetic remediation, the repair current is closely related to changes in soil resistance, and the soil resistance is a function of factors such as moisture content, salinity, and temperature [46]. Therefore, fluctuations in soil moisture content and increases in local temperature caused by current variations may also be significant factors contributing to changes in the repair current. During electrokinetic remediation, if the current is too high, a certain amount of heat is generated, leading to an increase in soil temperature. This elevated temperature can affect both electromigration and electroosmosis, thereby reducing the overall remediation efficiency.

Thus, in electrokinetic remediation experiments, selecting the optimal current density is crucial. This reduces the thermal effects caused by excessive current and helps improve the removal efficiency of contaminants, particularly heavy metals. By maintaining a balance between current density and temperature, the ion migration process can be optimized, enhancing the overall effectiveness of remediation. Hu and Cheng [47] studied the temperature variations in soil near the anode and cathode during electrokinetic remediation. The results showed that the soil temperatures near the anode and cathode increased to 26.9°C and 38.6°C, respectively, with a gradual increase in temperature from the anode to the cathode. This is because the soil resistance near the cathode increases, resulting in more Joule heat generation, which reduces the remediation efficiency.

In electrokinetic remediation, the soil moisture content must reach a certain level for the remediation effect to be significant. When the soil moisture content is too low, the remediation efficiency may be limited. Under a 3.0 V/cm electric field, the heat generated by the soil leads to a sharp decrease in soil moisture content, which in turn affects the remediation effect. However, studies have shown that the heat generated within the soil can help activate ions, increasing their migration under the influence of the electric field. Therefore, under such conditions, increasing the moisture content can enhance the remediation of saline soils.

Appropriately increasing the soil moisture content has a significant effect on the efficiency of electrokinetic remediation. However, once the moisture content reaches a certain threshold, further increases in moisture content have little effect on remediation efficiency. The conductivity of the soil medium varies with different moisture contents, which in turn affects the current density and, consequently, ion migration. Therefore, reaching a balanced moisture content is critical for optimizing the remediation efficiency in electrokinetic treatment [48].

Under electrification, soil moisture tends to migrate from the anode to the cathode. Especially at 3.0 V/cm, after 72 h of treatment, noticeable soil cracking was observed near the anode. At this point, the resistivity of the anode was relatively high, leading to a reduced remediation effect. Therefore, during remediation, it is beneficial to supplement the soil with water to maintain a sufficient moisture content, which can enhance the remediation efficiency.

A comparison of the data from the three experimental groups revealed that the optimal treatment condition for the electrokinetic remediation of salinized soils is 2.0 V/cm. Under these conditions, the removal efficiency of sodium ions (Na⁺) and chloride ions (Cl⁻) is relatively high (90.56% & 81.50%), while the removal efficiency of sulfate ions (SO₄²⁻) is lower (61.56%). Additionally, it is more challenging to remove anions from the soil than cations.

During the electrokinetic remediation of saline soil, the removal of sodium ions (Na^+^) and chloride ions (Cl^-^) was more effective than sulfate ions (SO_4_^2-^), whereas the removal of sulfate ions (SO_4_^2-^) was less efficient. Additionally, high voltages caused moisture loss near the electrodes, resulting in a sharp decrease in soil moisture content.

The relatively low removal rate of sulfate ions compared to sodium and chloride ions may be attributed to the following factors:First, the migration rate of ions in an electric field is inversely related to their hydrated ionic radius. Due to their higher charge density, sulfate ions attract more water molecules, forming a larger hydrated radius (approximately 0.4 nm), which is significantly greater than that of sodium or chloride ions. As a result, when moving through tortuous pore channels, sulfate ions experience increased viscous resistance and steric hindrance, leading to slower migration.Second, the doubly charged SO₄² ⁻ ions are subjected to electrostatic repulsion from negatively charged soil surfaces, making it difficult for them to diffuse into smaller pores. Additionally, they are more readily adsorbed and retained by positively charged sites in the soil, further reducing their effective migration rate.Third, under acidic conditions, SO₄² ⁻ tends to combine with calcium ions present in the soil to form calcium sulfate precipitate, which immobilizes the ions and prevents their migration.Fourth, all anions migrating toward the anode (including SO₄²⁻ and Cl⁻) counter a significant flow of hydrogen ions generated at the anode. This opposing movement impedes their transit, further hindering migration.

In electrochemical remediation, coarse-textured soils with low cation exchange capacity (CEC) facilitate rapid migration and removal of all ions. Conversely, fine-textured soils with high CEC significantly delay ion migration, particularly for cations like sodium (Na⁺). Their strong adsorption capacity creates an “ion reservoir” that requires more energy and time to clear, making the process more labor-intensive.

Considering the removal efficiencies of Na^+^, Cl^-^, and SO_4_^2-^ as well as energy consumption, the 2.0 V/cm) demonstrated the best overall performance under the experimental conditions. This group achieved the highest Na^+^ removal rate (90.56%), along with relatively high removal efficiencies for Cl^-^ (81.50%) and SO₄²⁻ (61.56%). Moreover, the total energy consumption was T3 group (3.0 V/cm, 570 Wh). Ion migration paths were also more defined, with enrichment primarily occurring near the electrodes, indicating good directionality and collectability.

In contrast, although the T1 group (1.0 V/cm) exhibited the lowest energy consumption (76.8 Wh), its removal efficiencies for Cl^-^ and SO_4_^2-^ were considerably lower. Notably, ion accumulation was observed in the S2 and S3 zones, suggesting that the electric field strength was insufficient to drive effective anion migration. While the T3 group (3.0 V/cm) showed a slight improvement in Cl^-^ removal, it experienced a decrease in Na^+^ removal efficiency (90.35%) and the highest energy consumption.

Therefore, the T2 group (2.0 V/cm) achieved the best balance among remediation efficiency, energy consumption, and ion enrichment behavior, making it the optimal electrokinetic treatment scheme for remediating saline loam soil in this study.

The pH values of the saline soil and electrolyte before and after treatment indicated the presence of both acidic and alkaline migration zones in the soil. Changes in pH can influence the electrokinetic remediation effect, and controlling pH is beneficial for ion migration in the soil.

Additionally, under a 3.0 V/cm electric field, the soil temperature increased, leading to a rapid decrease in soil moisture content, which, in turn, affected the remediation efficiency. However, this study also showed that the heat generated inside the soil can activate ions, increasing their migration under the influence of the electric field. Therefore, under these conditions, increasing the soil moisture content can improve the electrokinetic remediation of saline soils.

The study identified 2.0 V/cm as the optimal electric field strength for balancing removal efficiency and energy consumption in saline loam soils, thereby providing a reliable experimental basis for parameter selection in the electrokinetic remediation of salt-affected farmland. Furthermore, the findings demonstrate that a higher voltage does not necessarily lead to better performance. Instead, a moderate electric field intensity can maximize ion removal efficiency while minimizing soil moisture loss and energy consumption, offering important implications for the green and energy-efficient development of electrokinetic remediation technology.

The data related to this study are available in the Supporting Information files (S1–S3 Files).

## 5. Research recommendations and future outlook

(1) The experiments conducted in this study were laboratory-based and used artificially prepared saline soils. Future research should integrate relevant theoretical studies and field trials to assess the applicability and effectiveness of electrokinetic remediation technology in real-world engineering projects.
(2) This study focused solely on the electrokinetic remediation of saline‒alkali soils. Future work could explore the combination of this technique with other remediation methods in joint remediation experiments to further improve remediation efficiency.
(3) Research on soil heterogeneity and electrode durability under field conditions is crucial. Currently, we are conducting solar photovoltaic-based remediation studies in naturally severely saline soils to investigate how soil heterogeneity affects remediation efficacy. Simultaneously, we are evaluating the effectiveness of stainless steel electrode materials. The site layout has been completed, and we will strengthen research efforts in this area in subsequent studies.
(4) In this study, only the impact of electric field conditions was considered. Future research should further investigate the influence of additional factors and explore other enhanced electrokinetic remediation techniques to improve overall performance.
(5) This study adopted relatively small electrode spacing and low voltage settings based on the need for controllability under laboratory conditions, which differs from actual engineering applications. Future research should conduct large-scale field remediation experiments to better reflect real-world conditions.
(6) To further minimize energy consumption and reduce operational costs, future research may consider the integration of solar energy systems into electrokinetic remediation processes as a sustainable and cost-effective power supply alternative.

## Supporting information

S1 File Ion concentration matrix. (XLSX)

S2 File Energy consumption matrix. (XLSX)

S3 File Soil sample pH and electrolyte pH matrix. (XLSX)
